# Transmission cluster of cefiderocol-non-susceptible carbapenem-resistant Acinetobacter baumannii in cefiderocol-naïve individuals

**DOI:** 10.1186/s12941-024-00763-7

**Published:** 2024-11-29

**Authors:** Claudia Alteri, Antonio Teri, Maria Francesca Liporace, Antonio Muscatello, Leonardo Terranova, Margherita Carnevale Schianca, Federica Salari, Beatrice Silvia Orena, Flaminia Gentiloni Silverj, Mara Bernazzani, Simona Biscarini, Giulia Renisi, Lisa Cariani, Caterina Matinato, Ciro Canetta, Alessandra Bandera, Annapaola Callegaro

**Affiliations:** 1 Department of Oncology and Emato-Oncology, University of Milan, Milan, Italy; 2 Microbiology and Virology Unit, Fondazione IRCCS Ca’ Granda Ospedale Maggiore Policlinico, Milan, Italy; 3 Infectious Disease Unit, Fondazione IRCCS Ca’ Granda Ospedale Maggiore Policlinico, Milan, Italy; 4 Department of Internal Medicine, Respiratory Unit and Adult Cystic Fibrosis Center, Fondazione IRCCS Ca’ Granda Ospedale Maggiore Policlinico, Milan, Italy; 5 Residency in Microbiology and Virology Specialization School, University of Milan, Milan, Italy; 6 Medical Direction, Fondazione IRCCS Ca’ Granda Ospedale Maggiore Policlinico, Milan, Italy; 7 High Care Internal Medicine Unit, Fondazione IRCCS Ca’ Granda Ospedale Maggiore Policlinico, Milan, Italy; 8 Department of Pathophysiology and Transplantation, University of Milan, Milan, Italy

**Keywords:** Healthcare-associated infections, CRAB, Antimicrobial resistance, ESKAPEs, Surveillance, Cefiderocol

## Abstract

**Background:**

During prolonged FDC therapy, the emergence of FDC non-susceptibility in CRAB has been reported. Here, we report a transmission cluster of FDC-non-susceptible CRAB in four patients, all naïve to FDC treatment, characterized by a premature stop codon and amino acid deletion in the PirA protein.

**Methods:**

CRAB strains obtained from patients admitted in a single medicine ward of the IRCCS Fondazione Ospedale Maggiore Policlinico between March and July 2024 were analyzed by WGS and antimicrobial susceptibility testing. Phylogenetic analysis was used to assess their genetic relatedness.

**Results:**

Between March and July 2024, an outbreak of 33 CRAB was observed among hospitalized patients in a single ward at IRCCS. Genomic analysis, available in 29 cases, revealed that 24 isolates belonged to ST208/1806, 4 to ST369, and one to ST195/1816 (according to the Oxford scheme). FDC susceptibility was affected only in the four ST369 isolates (Kirby-Bauer disk diffusion diameter: 13 mm; UMIC^®^ method MIC: 4 mg/L), all characterized by a premature stop codon followed by a 52 amino acid deletion located between the amino acids 377 and 428 of the siderophore-drug receptor PirA. No other relevant mutations were detected in the iron-uptake genes. Core-genome ML tree including ST369 reference strains revealed that the four ST369 isolates were highly related and formed a distinct cluster (SNP distance: 3 [IQR: 1–6]). Of note, the four isolates were collected from four FDC-naïve individuals, two experiencing a CRAB-mediated infection.

**Conclusions:**

Our findings alert about the circulation of clones carrying modified siderophore-drug receptors without evidence of previous FDC treatment and support the importance of testing FDC susceptibility appropriately before its administration.

**Supplementary Information:**

The online version contains supplementary material available at 10.1186/s12941-024-00763-7.

## Background

Cefiderocol (FDC) is a new siderophore cephalosporin active against carbapenem-resistant Gram-negative bacteria [1]. The emergence of high-level resistance among multi-drug-resistant *Pseudomonas aeruginosa*, carbapenem-resistant *Enterobacterales* and *Acinetobacter baumannii* (CRAB) has been frequently reported after FDC exposure [2–4]. In *Pseudomonas aeruginosa* resistance to FDC has been described also in FDC-naïve individuals, or after ceftazidime/avibactam treatment [5, 6]. Overall, the resistance mechanisms are mainly mediated by alterations of siderophore receptors involved in FDC uptake. Mutations and/or deletions in the iron transport systems like PiuDC, or PiuA and PirA were strongly associated with increased FDC minimal inhibitory concentration (MIC) [7, 8]. Other resistance mechanisms like alterations in β-lactamase genes or mutations within the penicillin-binding protein PBP-3 can be associated with reduced FDC susceptibility [9].

Here we report the retrospective characterization of a transmission cluster sustained by a ST369 FDC-non-susceptible CRAB in FDC-naïve individuals.

## Methods

The present study characterizes four CRAB strains isolated from rectal (n = 1), groin (n = 1) blood culture (n = 1), and bronchoalveolar lavage (n = 1) of four patients, part of a CRAB outbreak emerging in a single medicine ward of the IRCCS Fondazione Ca’ Grande Ospedale Maggiore Policlinico, Milan, Italy between March and July 2024. In evaluating CRAB isolates, infections, and colonizations were defined according to Mangioni et al., 2023 [10].

Antimicrobial susceptibility testing was performed by broth microdilution for nine antibiotics as reported in Supplementary Text. FDC susceptibility was evaluated by Kirby-Bauer disk diffusion method, according to EUCAST guidelines v.14.0 [11]. FDC minimal inhibition concentration (MIC) was determined using the broth microdilution UMIC^®^ Cefiderocol (Bruker Daltonics, Bremen, Germany) on iron-depleted cation-adjusted Mueller Hinton broth. Antimicrobial susceptibility categorization was interpreted according to EUCAST guidelines v.14.0 [11]. For Acinetobacter baumannii, a clinical breakpoint for FDC has not yet been established. However, according to EUCAST guidelines [11], isolates with a zone diameter < 17 mm by disk diffusion should be considered non-susceptible.

CRAB genetic relatedness was evaluated by a combined approach using short-read whole genome sequence data and core alignment following the steps described in the Supplementary Text. A threshold of 10 single nucleotide polymorphisms (SNPs) was considered suggestive of the potential transmission cluster [10].

Ethical approval was not required because of the retrospective nature of this study based on bacterial isolates using aggregate clinical data.

## Results and discussion

Between March and July 2024, an outbreak of CRAB was observed among hospitalized patients in a single ward at IRCCS Fondazione Ospedale Maggiore Policlinico, Milan, Italy. Thirty-three patients (on 280 admissions) had at least one CRAB isolation during hospitalization (Supplementary Fig. 1). They were mainly male (25/33, 75.7%) with a median age of 79 years (interquartile range: 71–86). Of the 33 CRAB isolates, 29 were characterized for whole genome sequencing (WGS) according to Supplementary text [10]. The CRAB isolates represented an infection in 9/29 (31.0%) patients and a colonization in the remaining 20/29 (68.9%).

Genomic analysis of the 29 non-replicated CRAB collected during the outbreak revealed that all isolates belonged to the Pasteur sequence type 2 (ST2). According to the Oxford scheme, 24/29 (82.7%) isolates belonged to the ST208/1806, characterized by a capsular polysaccharide (K) “2” and a lipooligosaccharide outer core locus (OCL) “1”. Four isolates belonged to the ST369, characterized by the KL9 and OCL1. The remaining isolate belonged to ST195/1816, characterized by the KL3 and OCL1.

**Fig. 1:**
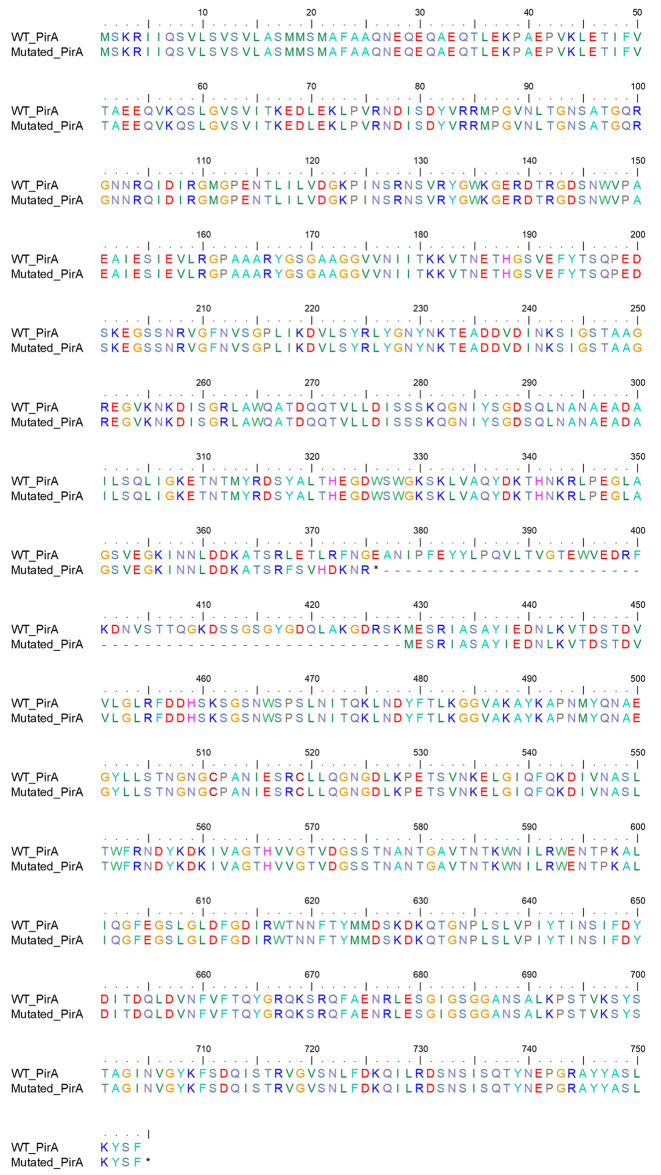
Amino acid alignment of the wild-type PirA and the mutated PirA, detected in the transmission chain of the four ST369 CRABs. An asterisk shows the premature stop codon. The deletion at amino acids 376–428 is shown by dashes

FDC susceptibility was affected only in the four ST369 isolates by the Kirby-Bauer disk diffusion (zone diameters: 13 mm) [11]. The commercial UMIC^®^ Cefiderocol (Bruker Daltonics, Bremen, Germany) method confirmed these results, assigning to the ST369 isolates a MIC equal to 4 mg/L. Of note, core genome analysis revealed the presence of a stop codon at amino acid position 376 preceded by a stretch of 8 amino acid mutations in the siderophore-drug receptor PirA in all four ST369 strains (E376stop), producing a premature signal to stop translation. Moreover, this stop codon is followed by a 52 amino acid deletion located between the amino acids 377 and 428 of PirA (Fig. 1). This deletion in the siderophore-drug receptor gene causes the loss of a domain involved in the binding of the siderophores, located at the amino acids 402–406 of the Acinetobacter baumannii PirA protein, and the complete and partial loss of the strand β9 and β8, respectively [12]. At the end of this deletion, at position 429 another Methionine was found, suggesting the production of a second truncated protein, containing the loops L7 and L11. As known, the binding site of siderophore receptors is formed by loops NL1, NL2, and NL3 of the plug domain and loops L3, L7, and L11 and strands β7, β8, and β9 of the β-barrel [12–15]. Thus, these alterations in PirA could result in truncated or highly altered proteins with poor functional siderophore receptor binding sites, and thus likely linked to weaker transportation of FDC and a decrease in FDC susceptibility. This is consistent with the FDC susceptibility results, defined by a zone diameter of 13 mm by disk diffusion and the MIC of 4 mg/L. SNP analyses using Snippy (https://github.com/tseemann/snippy) did not identify other relevant mutations in the iron-uptake genes with respect to ST369 reference sequences. Other amino acid variations in the efflux pump (EmrA/EmrK, *n* = 1), porin genes (OprD, *n* = 6), and penicillin-binding protein 1B (*n* = 1) were detected (Supplementary File 1).

The evaluation of clonal relatedness by a core-genome Maximum Likelihood (ML) tree including 22 ST369 reference strains (Supplementary Table 1) revealed that the four ST369 isolates were overall highly related (Supplementary Fig. 2, panel A) and formed a clear transmission cluster, characterized by a distance of 3 (IQR: 1–6) SNPs (Supplementary Fig. 2, panel B and C). All strains shared also a set of antimicrobial resistance and virulence genes, including the acquired carbapenemase gene *blaOXA-23*, the *blaOXA-66* (*OXA-51*-like variant) together with genes conferring resistance to aminoglycosides (*aph(3*′′*)-Ib* and *aph(6)-Id*), and tetracyclines (*tetA*), the cephalosporinase *ADC-30*, the multidrug efflux pumps of the resistance-nodulation-division (RND) family (*adeABC*, *adeFGHI*, and *adeJKL*), the Non-RND efflux systems *abeM* and *abeS*, the *sul2* sulphonamide resistance gene, as well as core virulence factors, usually displayed by *Acinetobacter baumannii* strains [16, 17]. The four ST369 isolates did not carry the *blaTEM* gene, the beta-lactamase *ADC-74*, the aminoglycoside-modifying enzyme *armA* gene, the macrolide resistance genes *mphE* and *msrE*, as well as the *pse*-pathway among the virulence genes. The antimicrobial resistance gene pathway described here was concordant with the MIC values obtained (Supplementary Table 2).

Regarding mobile genetic elements, all strains carried the plasmids tig00000534_pilon and pORAB01-2 (accession numbers: CP026708 and CP015485), and the prophage PHAGE_Acinet_Bphi_B1251_NC_019541.

**Fig. 2:**
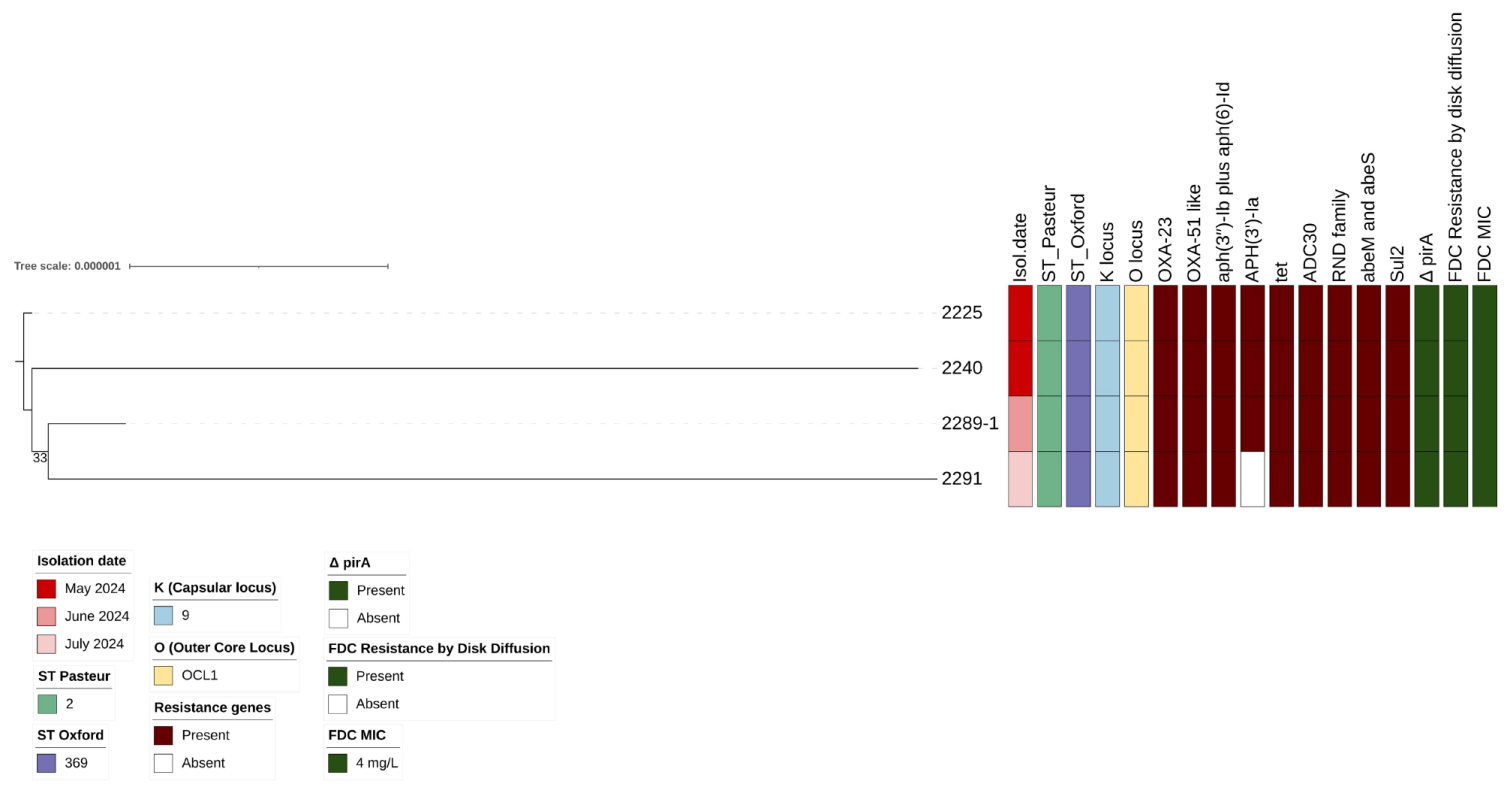
Estimated maximum likelihood phylogenetic analysis of the four Cefiderocol (FDC) non-susceptible carbapenem-resistant Acinetobacter baumannii isolates. The maximum likelihood was inferred from a core genome alignment of 3,344,425 bp. The phylogeny was estimated with IqTree using the best-fit model of nucleotide substitution HKY + F with 1,000 replicates and fast bootstrapping. The numbers on leaves represent the sample IDs, and bootstrap values are shown on branches. Information regarding the samples was reported: date of isolation, the sequence type (ST), capsular locus (K locus) and lipooligosaccharide outer core (OC locus), the presence (solid squares) or absence of antimicrobial resistance genes, the presence of deletion in *pirA* gene, the Cefiderocol-non-susceptibility, tested by disk diffusion (according to the last EUCAST guidelines, https://www.eucast.org/fileadmin/src/media/PDFs/EUCAST_files/Breakpoint_tables/v_14.0_Breakpoint_Tables.pdf) and the minimal inhibitory concentration (MIC) value by the UMIC (Bruker) commercial test

Surprisingly, all the ST369 isolates were collected from FDC naïve individuals admitted to the medicine ward from May to July 2024 (Fig. 2). All individuals but one received previous antimicrobial therapy. Unfortunately, no information is available regarding the antimicrobial drugs given in the months preceding CRAB detection, which could have contributed to lower susceptibility to FDC.

Two of the four patients experienced a CRAB-mediated infection. One was treated with an initial FDC-containing regimen; clinical conditions did not improve since the administration of alternative antimicrobial treatment (clinical case manuscript is in progress).

The first ST369 case was isolated in May in a patient admitted to the ward a few days before, without CRAB surveillance being performed at their admittance. Thus, a CRAB acquisition outside the medicine ward cannot be excluded. In the remaining three cases, CRAB at patients’ admission was negative and its isolation occurred more than 48 h from admission, suggesting their acquisition inside the medicine ward.

A modest rate of evolution was observed when the isolates were analyzed alone without reference genomes (Fig. 2). By this analysis, modest genomic divergence can be observed in ID2291 with respect to the other isolates, due to the acquisition of 19 new SNPs and three deletions (Supplementary File 2), and the loss of the chromosomal-encoded aminoglycoside phosphotransferase *aph(3’-Ia)* gene. This is consistent with the time of acquisition of CRAB in this patient, occurring two months after the first putative case.

As a limitation of our findings, to define the MIC for FDC we used a commercially available test on iron-depleted cation-adjusted Mueller Hinton broth microdilution not yet recommended by EUCAST [18]. A recent paper that evaluated the performances of commercial tests in testing FDC activity [19], showed that the test we used produced few very major errors with respect to the other MIC-based methods and exhibited the highest essential agreement with the standard broth microdilution method. Moreover, this MIC-based test was used in combination with the disk diffusion method, recommended by the EUCAST guidelines v.14.0 [11]. Both tests were concordant in the definition of FDC-non-susceptibility. As a further limitation, we cannot speculate about the event that caused the siderophore-drug receptor PirA alteration, like previous non-FDC antimicrobial treatment exposures, as described in *Pseudomonas aeruginosa* [5, 6], or other selection mechanisms. Indeed, even if three out of the four received previous antimicrobial therapy, information about the class and type of antimicrobial treatment is unavailable. Information about potential transmission pathways is limited and the environment was not sampled for CRAB presence.

Finally, even though the alterations detected in PirA can suggest a role in affecting the susceptibility to FDC, the perfect correlation between changes in siderophores systems and FDC MICs remains to be defined [20], especially when there may be confounding factors such as levels of expression of beta-lactamases (i.e. AmpC, OXA-23, or OXA-66) or efflux pumps.

## Conclusion

We report a transmission cluster of an FDC-non-susceptible ST369 CRAB strain in the absence of FDC treatment, characterized by a premature stop codon and a large genomic deletion in the PirA protein. These findings alert about the circulation and cross-transmission of clones carrying specific FDC-non-susceptibility mechanisms without evidence of previous FDC treatment. Thus, monitoring the CRAB outbreaks by whole genome sequencing is urgent and will help mitigate the emergence and spread of non-susceptible strains to this new siderophore cephalosporin. These results also support the importance of testing the FDC susceptibility appropriately before its administration and adopting routine infection prevention and control practices, including screening for CRAB at admission and every week.

## Electronic supplementary material

Below is the link to the electronic supplementary material.

Supplementary Material 1

Supplementary Material 2

Supplementary Material 3

Supplementary Material 4

Supplementary Material 5

Supplementary Material 6

Supplementary Material 7

## Data Availability

The CRAB sequences obtained in this study are openly available on the SRA portal under the accession numbers SAMN43144136-SAMN43144139 (BioProject: PRJNA1147175). This published article and its supplementary information files include all the other data analyzed during this study.

## Abbreviations

FDC: Cefiderocol
CRAB: Carbapenem-resistant Acinetobacter baumannii
MIC: Minimal inhibitory concentration
SNP: Single nucleotide polymorphism
WGS: Whole genome sequencing
K: Capsular polysaccharide
OCL: Lipooligosaccharide outer core
ML: Maximum likelihood
