# Supplementary material for: Transmission cluster of cefiderocol-non-susceptible carbapenem-resistant Acinetobacter baumannii in cefiderocol-naïve individuals
Source: Ann Clin Microbiol Antimicrob. 2024 Nov 29;23:104. doi: 10.1186/s12941-024-00763-7 (PMC11607823; doi:10.1186/s12941-024-00763-7)
Supplement: Supplementary file 1 — Supplementary Material 1 [file 12941_2024_763_MOESM1_ESM.docx]

**Supplementary Text**

**Materials and Methods**

**CRAB identification and antimicrobial susceptibility testing**

Strains were identified by matrix-assisted laser desorption/ionization time-of-flight mass spectrometry (MALDI-TOF/MS, VITEK® MS PRIME, bioMérieux), and screened for the presence of OXA-23-like, OXA-24/58-like, and NDM carbapenemases using a lateral flow assay (Coris RESIST Acineto, Coris bioConcept, Gembloux, Belgium). Antimicrobial susceptibility testing was performed by broth microdilution using the Microscan WalkAway (Beckman Coulter, Inc.) for nine antibiotics (amikacin, ciprofloxacin, gentamicin, imipenem, levofloxacin, meropenem, trimethoprim-sulfamethoxazole, tobramycin, and colistin). To evaluate susceptibility to FDC, the Kirby-Bauer disk diffusion method was performed on CAMH-agar plates (BioMérieux, Marcy L’Etoile, France) according to EUCAST guidelines v.14.0 [1]. The minimum inhibitory concentration (MIC) of FDC was determined using the broth microdilution UMIC® Cefiderocol (Bruker Daltonics, Bremen, Germany) on ID-CA-MHB (iron-depleted cation-adjusted Mueller Hinton broth). Susceptible and resistant categories were assigned according to the EUCAST breakpoint table (v.14.0 [1]; available at [https://www.eucast.org/clinical_breakpoints](https://www.eucast.org/clinical_breakpoints)). Regarding cefiderocol, even if EUCAST has not yet established clinical breakpoints for CRAB, according to EUCAST guidelines [1], isolates with a zone diameter <17 mm by disk diffusion should be considered non-susceptible.

**Whole genome sequencing (WGS)**

Bacterial DNA was extracted from CRAB pure isolates using ZymoBIOMICS DNA Miniprep Kit (Zymo Reseach) in accordance with manufacturer's instructions. Libraries for whole genome sequencing were generated using Illumina DNA Library Prep kit (Illumina, Inc., San Diego, CA, USA). PCR products were visualized with Bioanalyzer 2100 and High Sensitivity DNA kit (Agilent, Santa Clara, CA, USA). Finally, libraries were sequenced on Illumina MiSeq sequencing platform (Illumina, San Diego, CA, USA) using MiSeq Reagent Kit v2.

**Data analysis**

Genomic data were assembled as described in [2]. Briefly, a multilocus sequence typing (MLST) was performed with the mlst tool (v.2.11) [3,4], using the Pasteur and Oxford scheme [5,6]. The capsular polysaccharide loci (KL) and outer core lipooligosaccharide loci (OCL) were assessed by Kaptive (v.0.7.3) [7]. Investigation of antibiotic resistance (AMR) genes was carried out with ABRicate (v.0.4), by using the Comprehensive Antibiotic Resistance Database (CARD) [8] and ResFinder database [9], while virulence factors were investigated using the Virulence Factor Database (VFDB) [10]. The MobileElementFinder tool (v.1.0.3) [11], was used for the identification of mobile genetic elements (MGEs), while the presence of intact bacteriophages was investigated using PHASTER [12,13]. Plasmids were inferred from contigs using the MOB suite tool [14]. All these analyses were performed on the whole genome.

**Core genome and phylogenetic analysis**

To explore the concordance between the predicted ST and the four CRAB isolates, and to define the genetic relatedness of the four CRAB isolates, a Maximum Likelihood (ML) tree based on a core genome alignment obtained by 22 ST369 reference sequences and the four CRAB isolates was performed. Core genome analysis was performed with Roary (v.3.13.0) [15], with default parameters, obtaining a core genome alignment shared by 95% of the isolates. This core genome alignment was then inspected by maximum likelihood (ML) and minimum spanning tree (MST) methods. The ML tree was constructed by IQTREE (v.2.0.6) [16] with 1000 bootstrap replicates under the best nucleotide substitution model TIM+F+I+G4, inferred by Model Finder [17]. Phylogenetic trees were visualized and annotated using iTOL (v5) [18]. Minimum Spanning trees were constructed on the core genome alignment with Grapetree (v1.5.0) [19] and pairwise SNP distances were calculated using snp-dists tool (<https://github.com/tseemann/snp-dists>). A threshold of 10 SNPs was considered suggestive of the potential transmission cluster [2]. Variant calling was performed using snippy (<https://github.com/tseemann/snippy>) using CP091345 as a reference.

**References**

1. Eucast guidelines v.14.0 Available at <https://www.eucast.org/fileadmin/src/media/PDFs/EUCAST_files/Breakpoint_tables/v_14.0_Breakpoint_Tables.pdf>

2. Mangioni D, Fox V, Chatenoud L, Bolis M, Bottino N, Cariani L, Gentiloni Silverj F, Matinato C, Monti G, Muscatello A, Teri A, Terranova L, Piatti A, Gori A, Grasselli G, Stocchetti N, Alteri C, Bandera A. 2024. Genomic Characterization of Carbapenem-Resistant Acinetobacter baumannii (CRAB) in Mechanically Ventilated COVID-19 Patients and Impact of Infection Control Measures on Reducing CRAB Circulation during the Second Wave of the SARS-CoV-2 Pandemic in Milan, Italy. Microbiol Spectr. 11(2):e0020923. doi: 10.1128/spectrum.00209-23.

3. Jolley KA, Maiden MC. 2010. BIGSdb: scalable analysis of bacterial genome variation at the population level. *BMC Bioinformatics* 11:595. doi: 10.1186/1471-2105-11-595]

4. Seemann T. 2022. mlst tool. [https://github.com/tseemann/mlst](https://github.com/tseemann/mlst).

5. Diancourt L, Passet V, Nemec A, Dijkshoorn L, Brisse S. 2010. The population structure of Acinetobacter baumannii: expanding multiresistant clones from an ancestral susceptible genetic pool. *PLoS One* 5:e10034. doi: 10.1371/journal.pone.0010034

6. Bartual SG, Seifert H, Hippler C, Luzon MA, Wisplinghoff H, Rodríguez-Valera F. 2005. Development of a multilocus sequence typing scheme for characterization of clinical isolates of Acinetobacter baumannii. *J Clin Microbiol* 43:4382–4390. doi: 10.1128/JCM.43.9.4382-4390.2005

7. Wyres KL, Cahill SM, Holt KE, Hall RM, Kenyon JJ. 2020. Identification of Acinetobacter baumannii loci for capsular polysaccharide (KL) and lipooligosaccharide outer core (OCL) synthesis in genome assemblies using curated reference databases compatible with Kaptive. Microb Genom 6:e000339. doi: 10.1099/mgen.0.000339

8. Jia B, Raphenya AR, Alcock B, Waglechner N, Guo P, Tsang KK, Lago BA, Dave BM, Pereira S, Sharma AN, Doshi S, Courtot M, Lo R, Williams LE, Frye JG, Elsayegh T, Sardar D, Westman EL, Pawlowski AC, Johnson TA, Brinkman FSL, Wright GD, McArthur AG. 2017. CARD 2017: expansion and model-centric curation of the comprehensive antibiotic resistance database. *Nucleic Acids Res* 45:D566–D573. doi: 10.1093/nar/gkw1004

9. Zankari E, Hasman H, Cosentino S, Vestergaard M, Rasmussen S, Lund O, Aarestrup FM, Larsen MV. 2012. Identification of acquired antimicrobial resistance genes. *J Antimicrob Chemother* 67:2640–2644. doi: 10.1093/jac/dks261

10. Chen L, Zheng D, Liu B, Yang J, Jin Q. 2016. VFDB 2016: hierarchical and refined dataset for big data analysis—10 years on. *Nucleic Acids Res* 44:D694–D697. doi: 10.1093/nar/gkv1239

11. Johansson MHK, Bortolaia V, Tansirichaiya S, Aarestrup FM, Roberts AP, Petersen TN. 2021. Detection of mobile genetic elements associated with antibiotic resistance in Salmonella enterica using a newly developed web tool: MobileElementFinder. J Antimicrob Chemother 76:101–109. doi: 10.1093/jac/dkaa390

12. Zhou Y, Liang Y, Lynch KH, Dennis JJ, Wishart DS. 2011. PHAST: a fast phage search tool. *Nucleic Acids Res* 39:W347–W352. doi: 10.1093/nar/gkr485.

13. Arndt D, Grant JR, Marcu A, Sajed T, Pon A, Liang Y, Wishart DS. 2016. PHASTER: a better, faster version of the PHAST phage search tool. *Nucleic Acids Res* 44:W16–W21. doi: 10.1093/nar/gkw387

14. Robertson J, Nash JHE. 2018. MOB-suite: software tools for clustering, reconstruction and typing of plasmids from draft assemblies. Microb Genom 4:e000206. doi: 10.1099/mgen.0.000206.

15. Page AJ, Cummins CA, Hunt M, Wong VK, Reuter S, Holden MTG, Fookes M, Falush D, Keane JA, Parkhill J. 2015. Roary: rapid large-scale prokaryote pan genome analysis. *Bioinformatics* 31:3691–3693. doi: 10.1093/bioinformatics/btv421

16. Nguyen LT, Schmidt HA, von Haeseler A, Minh BQ. 2015. IQ-TREE: a fast and effective stochastic algorithm for estimating maximum-likelihood phylogenies.. Mol Biol Evol 32(1):268-274 7.

17. Kalyaanamoorthy S, Minh BQ, Wong TKF, von Haeseler A, Jermiin LS. 2017. ModelFinder: fast model selection for accurate phylogenetic estimates. Nat Methods14(6):587-589

18. Letunic I, Bork P. 2021. Interactive Tree Of Life (iTOL) v5: an online tool for phylogenetic tree display and annotation. Nucleic Acids Res 49(W1):W293-W296

19. Zhou Z, Alikhan NF, Sergeant MJ, Luhmann N, Vaz C, Francisco AP, Carriço JA, Achtman M. GrapeTree: visualization of core genomic relationships among 100,000 bacterial pathogens. Genome Res. 2018 Sep;28(9):1395-1404. doi: 10.1101/gr.232397.117.
