# Supplementary material for: Transmission cluster of cefiderocol-non-susceptible carbapenem-resistant Acinetobacter baumannii in cefiderocol-naïve individuals
Source: Ann Clin Microbiol Antimicrob. 2024 Nov 29;23:104. doi: 10.1186/s12941-024-00763-7 (PMC11607823; doi:10.1186/s12941-024-00763-7)
Supplement: Supplementary file 2 — Supplementary Material 2 [file 12941_2024_763_MOESM2_ESM.pdf]

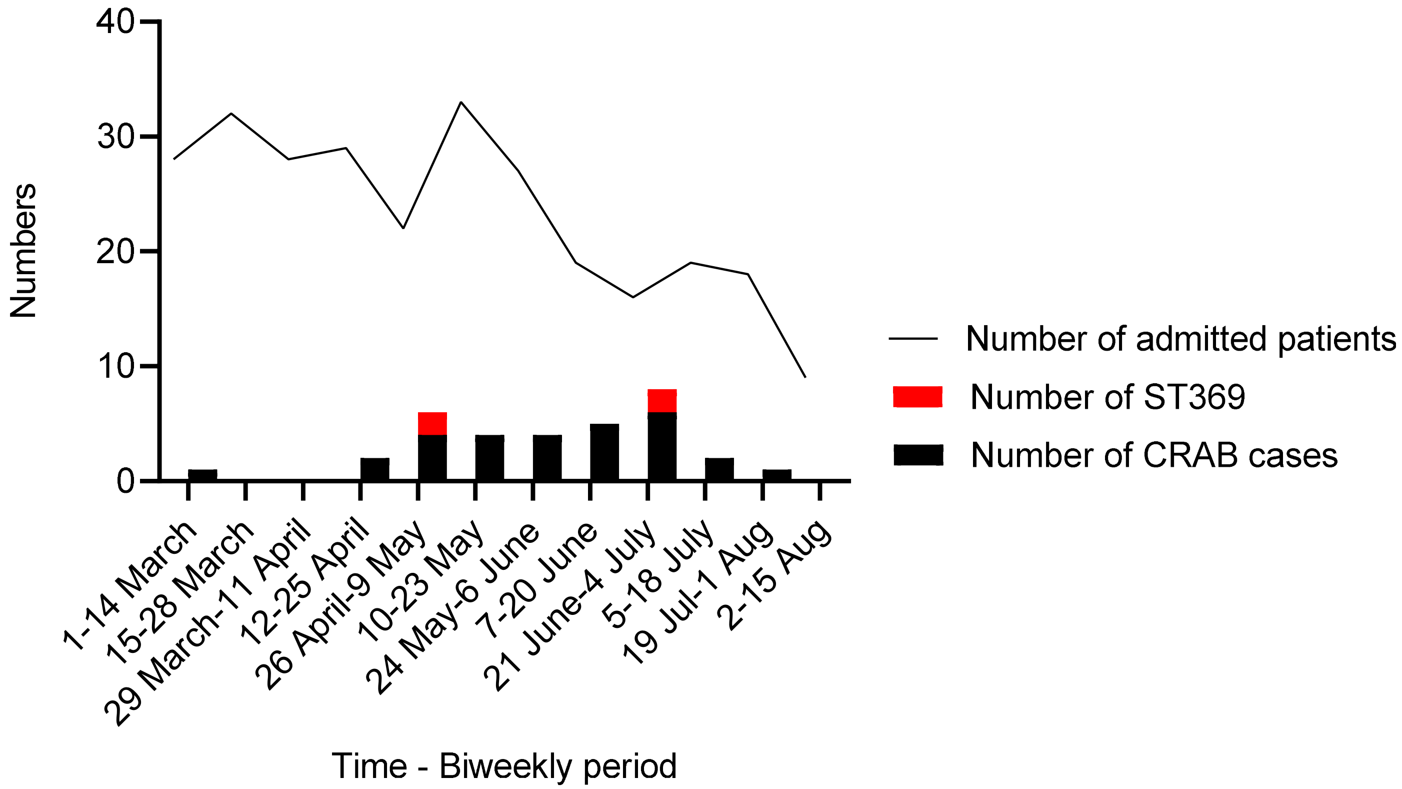

Supplementary Figure 1. Temporal trend by fifteen days of the 33 microbiologically-confirmed carbapenem-resistant Acinetobacter baumannii (CRAB) cases between March and August 2024 with respect to the number of admitted patients in the ward. The four ST369 cases were highlighted in red.
