# Supplementary material for: Transmission cluster of cefiderocol-non-susceptible carbapenem-resistant Acinetobacter baumannii in cefiderocol-naïve individuals
Source: Ann Clin Microbiol Antimicrob. 2024 Nov 29;23:104. doi: 10.1186/s12941-024-00763-7 (PMC11607823; doi:10.1186/s12941-024-00763-7)
Supplement: Supplementary file 3 — Supplementary Material 3 [file 12941_2024_763_MOESM3_ESM.pdf]

A

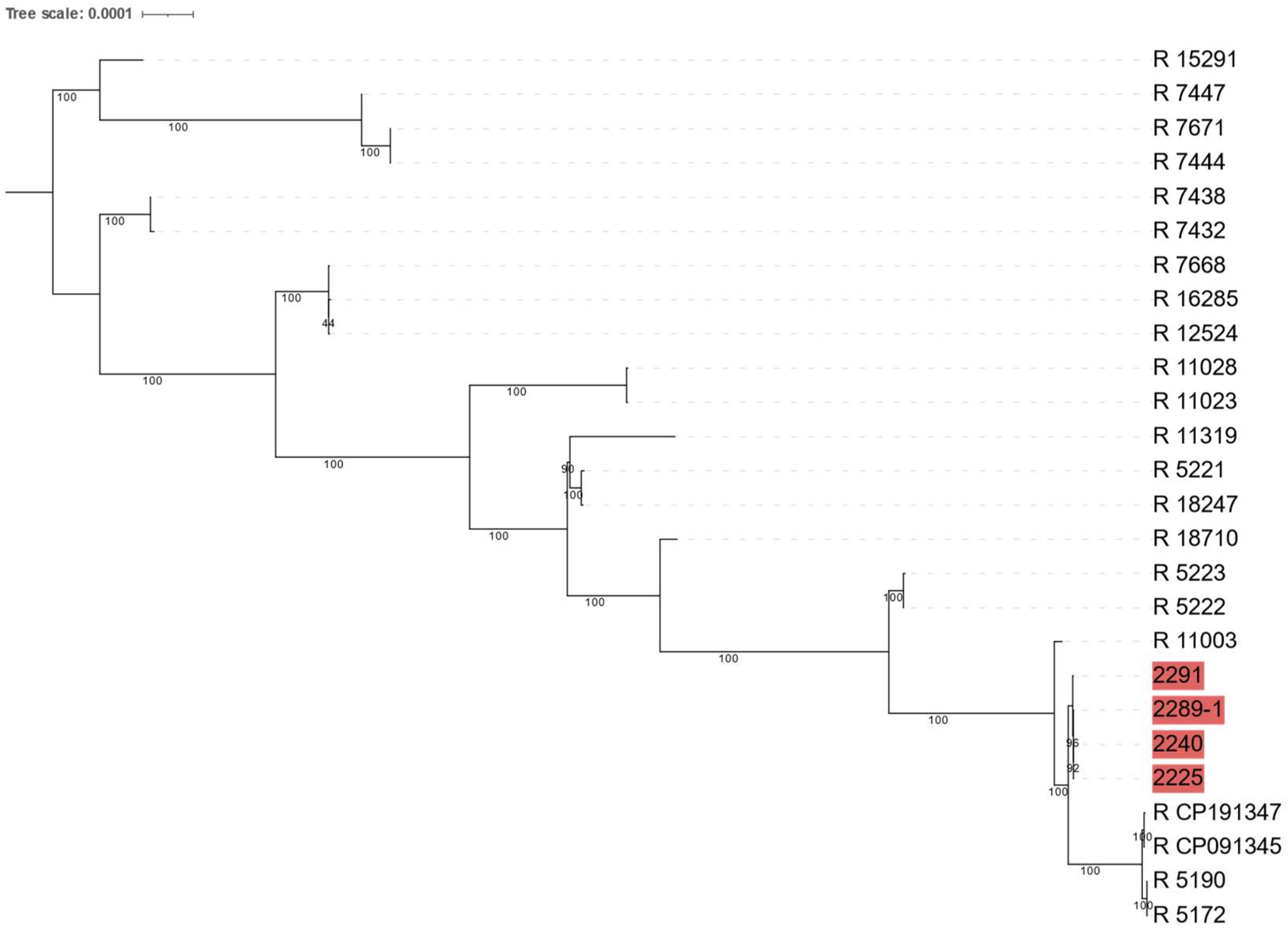
Tree scale: 0.0001
R 15291
R 7447
R 7671
R 7444
R 7438
R 7432
R 7668
R 16285
R 12524
R 11028
R 11023
R 11319
R 5221
R 18247
R 18710
R 5223
R 5222
R 11003
2291
2289-1
2240
2225
R CP191347
R CP091345
R 5190
R 5172

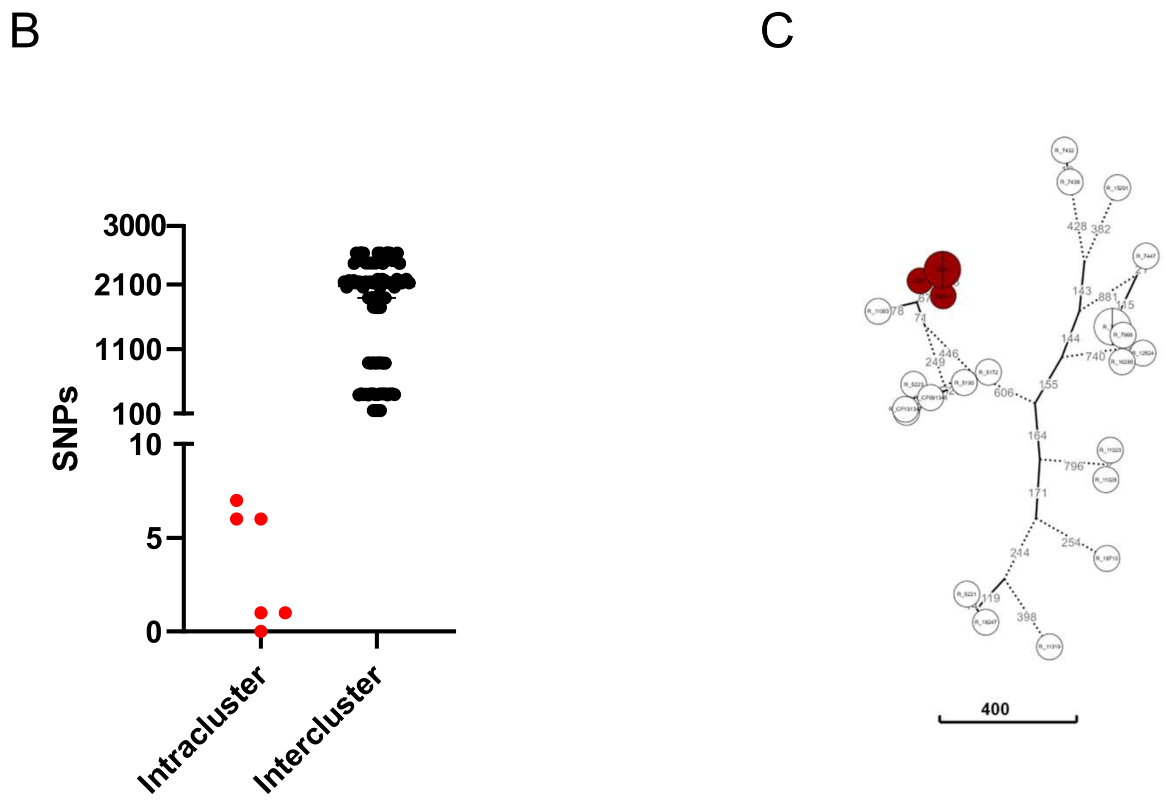

Supplementary Figure 2. Genetic relatedness of the four ST369 CRAB strains.
A. Estimated Maximum Likelihood phylogenetic analysis of the four cefiderocol-non-susceptible carpabenem resistant ST369 Acinetobacter baumannii isolated from May 2024 to July 2024 in a single ward and ST369 reference genomes (*N*=22). The Maximum Likelihood tree was inferred from a core-genome alignment of 2,435,110 bp. The phylogeny was estimated with IqTree using the best-fit model of nucleotide substitution TIM+F+I+G4 with 1,000 replicates and fast bootstrapping. The numbers on the leaves represent the sample IDs. The four isolates characterizing the ST369 transmission cluster are highlighted in red. Bootstrap values are displayed on branches. **B.** Scatter plot showing the intra- and inter-cluster distances for the Maximum Likelihood (ML) ST369 cluster. The SNP distances among the four isolates characterizing the ST369 transmission cluster are shown in red. **C.** Minimum Spanning Tree. The four isolates characterizing the ST369 transmission cluster are shown in red.
