# Supplementary material for: Transmission cluster of cefiderocol-non-susceptible carbapenem-resistant Acinetobacter baumannii in cefiderocol-naïve individuals
Source: Ann Clin Microbiol Antimicrob. 2024 Nov 29;23:104. doi: 10.1186/s12941-024-00763-7 (PMC11607823; doi:10.1186/s12941-024-00763-7)
Supplement: Supplementary file 4 — Supplementary Material 4 [file 12941_2024_763_MOESM4_ESM.docx]

**Supplementary Table 1:** Acinetobacter baumannii ST369 reference genomes included in phylogenetic analysis.

| **ID** | **Isolate** | **Country** | **Year** | **Species** | **ST_OXFORD** | **Database** |
| --- | --- | --- | --- | --- | --- | --- |
| [5172](https://pubmlst.org/bigsdb?page=info&db=pubmlst_abaumannii_isolates&id=5172) | 51211 | China | 2014 | Acinetobacter baumannii | 369;1837 | pubmlst.org |
| [5190](https://pubmlst.org/bigsdb?page=info&db=pubmlst_abaumannii_isolates&id=5190) | 130911 | China | 2014 | Acinetobacter baumannii | 369;1837 | pubmlst.org |
| [5221](https://pubmlst.org/bigsdb?page=info&db=pubmlst_abaumannii_isolates&id=5221) | 173189 | China | 2017 | Acinetobacter baumannii | 369;1837 | pubmlst.org |
| [5222](https://pubmlst.org/bigsdb?page=info&db=pubmlst_abaumannii_isolates&id=5222) | 173329 | China | 2017 | Acinetobacter baumannii | 369;1837 | pubmlst.org |
| [5223](https://pubmlst.org/bigsdb?page=info&db=pubmlst_abaumannii_isolates&id=5223) | 173625 | China | 2017 | Acinetobacter baumannii | 369;1837 | pubmlst.org |
| [7432](https://pubmlst.org/bigsdb?page=info&db=pubmlst_abaumannii_isolates&id=7432) | 472-03 | Mexico | 2011 | Acinetobacter baumannii | 369;1837 | pubmlst.org |
| [7438](https://pubmlst.org/bigsdb?page=info&db=pubmlst_abaumannii_isolates&id=7438) | 521-03 | Mexico | 2011 | Acinetobacter baumannii | 369;1837 | pubmlst.org |
| [7444](https://pubmlst.org/bigsdb?page=info&db=pubmlst_abaumannii_isolates&id=7444) | 097-16 HCG | Mexico | 2016 | Acinetobacter baumannii | 369;1837 | pubmlst.org |
| [7447](https://pubmlst.org/bigsdb?page=info&db=pubmlst_abaumannii_isolates&id=7447) | 156-16 HCG | Mexico | 2016 | Acinetobacter baumannii | 369;1837 | pubmlst.org |
| [7668](https://pubmlst.org/bigsdb?page=info&db=pubmlst_abaumannii_isolates&id=7668) | 25-176-2015 HRGIZ | Mexico | 2015 | Acinetobacter baumannii | 369;1837 | pubmlst.org |
| [7671](https://pubmlst.org/bigsdb?page=info&db=pubmlst_abaumannii_isolates&id=7671) | 185-16 HCG | Mexico | 2016 | Acinetobacter baumannii | 369;1837 | pubmlst.org |
| [11003](https://pubmlst.org/bigsdb?page=info&db=pubmlst_abaumannii_isolates&id=11003) | SAMEA5229256 | Italy | 2018 | Acinetobacter baumannii | 369;1837 | pubmlst.org |
| [11023](https://pubmlst.org/bigsdb?page=info&db=pubmlst_abaumannii_isolates&id=11023) | SAMEA5396108 | Germany | 2014 | Acinetobacter baumannii | 369;1837 | pubmlst.org |
| [11028](https://pubmlst.org/bigsdb?page=info&db=pubmlst_abaumannii_isolates&id=11028) | SAMEA5396113 | Germany | 2014 | Acinetobacter baumannii | 369;1837 | pubmlst.org |
| [11319](https://pubmlst.org/bigsdb?page=info&db=pubmlst_abaumannii_isolates&id=11319) | SAMEA9273293 | India | 2019 | Acinetobacter baumannii | 369;1837 | pubmlst.org |
| [12524](https://pubmlst.org/bigsdb?page=info&db=pubmlst_abaumannii_isolates&id=12524) | SAMN15907185 | USA | 2020 | Acinetobacter baumannii | 369;1837 | pubmlst.org |
| [15291](https://pubmlst.org/bigsdb?page=info&db=pubmlst_abaumannii_isolates&id=15291) | SAMN21493826 | USA | 2021 | Acinetobacter baumannii | 369;1837 | pubmlst.org |
| [16285](https://pubmlst.org/bigsdb?page=info&db=pubmlst_abaumannii_isolates&id=16285) | SAMN24300475 | USA | 2021 | Acinetobacter baumannii | 369;1837 | pubmlst.org |
| [18247](https://pubmlst.org/bigsdb?page=info&db=pubmlst_abaumannii_isolates&id=18247) | SAMN19762789 | China | 2019 | Acinetobacter baumannii | 369;1837 | pubmlst.org |
| [18710](https://pubmlst.org/bigsdb?page=info&db=pubmlst_abaumannii_isolates&id=18710) | 90927 | China | 2023 | Acinetobacter baumannii | 369;1837 | pubmlst.org |
| CP091347 | SAMN25131663 | Belgium | 2022 | Acinetobacter baumannii | 369 | NCBI |
| CP091345 | SAMN25131665 | Belgium | 2022 | Acinetobacter baumannii | 369 | NCBI |
