# Supplementary material for: Transmission cluster of cefiderocol-non-susceptible carbapenem-resistant Acinetobacter baumannii in cefiderocol-naïve individuals
Source: Ann Clin Microbiol Antimicrob. 2024 Nov 29;23:104. doi: 10.1186/s12941-024-00763-7 (PMC11607823; doi:10.1186/s12941-024-00763-7)
Supplement: Supplementary file 5 — Supplementary Material 5 [file 12941_2024_763_MOESM5_ESM.docx]

**Supplementary Table 2:** Minimal inhibitory concentration (MIC, expressed as mg/L) values of the tested antibiotics.

| **Sample** | **AMK** | | **CIP** | | **GEN** | | **IPM** | | **LVX** | | **MEM** | | **STX** | | **TIS** | | **COL** | | | **FDC** | | |
| --- | --- | --- | --- | --- | --- | --- | --- | --- | --- | --- | --- | --- | --- | --- | --- | --- | --- | --- | --- | --- | --- | --- |
|  | **MIC** | **Interp (S≤8)** | **MIC** | **Interp (S≤0.001)** | **MIC** | **Interp (S≤4)** | **MIC** | **Interp (S≤2)** | **MIC** | **Interp (S≤0.5)** | **MIC** | **Interp (S≤)** | **MIC** | **Interp (S≤2)** | **MIC** | **Interp (S≤4)** | | **MIC** | **Interp (S≤2)** | **Disk mm** | **MIC** | **Interp^a^** |
| 2240 | <=8 | S | >1 | R | <=2 | S | >4 | R | >1 | R | 32 | R | >4/76 | R | <=2 | S | | 1 | S | 13 | 4 | NS |
| 2289-1 | <=8 | S | >1 | R | <=2 | S | >4 | R | >1 | R | 32 | R | >4/76 | R | <=2 | S | | 1 | S | 13 | 4 | NS |
| 2291 | <=8 | S | >1 | R | <=2 | S | >4 | R | >1 | R | 32 | R | >4/76 | R | <=2 | S | | 2 | S | 13 | 4 | NS |
| 2225 | <=8 | S | >1 | R | <=2 | S | >4 | R | >1 | R | 32 | R | >4/76 | R | <=2 | S | | 0.75 | S | 13 | 4 | NS |

Abbreviations: Interp=Interpretation, AMK=amikacin, CIP=ciprofloxacin, GEN=gentamicin, IPM=imipenem, LVX=levofloxacin, MEM=meropenem, SXT=trimethoprim-sulfamethoxazole, TIS=tobramycin, CST=colistin, FDC=cefiderocol, S=susceptible, R=resistance. Susceptible and resistant categories were assigned according to the EUCAST breakpoint table (version 14.0, available at <https://www.eucast.org/fileadmin/src/media/PDFs/EUCAST_files/Breakpoint_tables/v_14.0_Breakpoint_Tables.pdf>). **^a^**For Acinetobacter baumannii, a clinical breakpoint for FDC has not yet been established. However, according to EUCAST guidelines (version 14.0, available at <https://www.eucast.org/fileadmin/src/media/PDFs/EUCAST_files/Breakpoint_tables/v_14.0_Breakpoint_Tables.pdf>), isolates with a zone diameter <17 mm by disk diffusion should be considered non-susceptible (NS).
